# Enhanced transgene expression in rice following selection controlled by weak promoters

**DOI:** 10.1186/1472-6750-13-29

**Published:** 2013-03-27

**Authors:** Jie Zhou, Yong Yang, Xuming Wang, Feibo Yu, Chulang Yu, Juan Chen, Ye Cheng, Chenqi Yan, Jianping Chen

**Affiliations:** 1 State Key Laboratory Breeding Base for Zhejiang Sustainable Pest and Disease Control, MOA Key Laboratory for Plant Protection and Biotechnology, Zhejiang Provincial Key Laboratory of Plant Virology, Zhejiang Academy of Agricultural Sciences, Hangzhou, 310021, P.R.China

**Keywords:** tCUP, Promoter activity, Selectable marker, Transgene expression, Copy number, Rice

## Abstract

**Background:**

Techniques that enable high levels of transgene expression in plants are attractive for the commercial production of plant-made recombinant pharmaceutical proteins or other gene transfer related strategies. The conventional way to increase the yield of desired transgenic products is to use strong promoters to control the expression of the transgene. Although many such promoters have been identified and characterized, the increase obtainable from a single promoter is ultimately limited to a certain extent.

**Results:**

In this study, we report a method to magnify the effect of a single promoter by using a weak promoter-based selection system in transgenic rice. tCUP1, a fragment derived from the tobacco cryptic promoter (tCUP), was tested for its activity in rice by fusion to both a β-glucuronidase (GUS) reporter and a hygromycin phosphotransferase (HPT) selectable marker. The tCUP1 promoter allowed the recovery of transformed rice plants and conferred tissue specific expression of the GUS reporter, but was much weaker than the CaMV 35S promoter in driving a selectable marker for growth of resistant calli. However, in the resistant calli and regenerated transgenic plants selected by the use of tCUP1, the constitutive expression of green fluorescent protein (GFP) was dramatically increased as a result of the additive effect of multiple T-DNA insertions. The correlation between attenuated selection by a weak promoter and elevation of copy number and foreign gene expression was confirmed by using another relatively weak promoter from nopaline synthase (Nos).

**Conclusions:**

The use of weak promoter derived selectable markers leads to a high T-DNA copy number and then greatly increases the expression of the foreign gene. The method described here provides an effective approach to robustly enhance the expression of heterogenous transgenes through copy number manipulation in rice.

## Background

Plant biotechnology has great potential to speed up crop improvement and meet the needs of a growing world population. A wide variety of commercial crops with important agronomic traits have already been produced successfully. Similar methods have also made it possible to use plants as bioreactors for economic production of pharmaceuticals. Although various plant expression systems have been established as bioreactors, the wider use of plant-derived pharmaceuticals has been limited by low yields due to the dozens of important parameters critically reviewed by Egelkrout et al. [1].

Various strategies to maximize transgene expression have been investigated to meet the demands of fundamental research or industrial applications. Employing a strong promoter for transcription is the first method of choice. The most widely used constitutive promoter for transgene expression in plants is the 35S promoter of cauliflower mosaic virus (CaMV) [2,3]. The use of tandem repeats of the fragment between positions -343 and -90 (the enhanced CaMV 35S promoter) enhances the level of promoter activity up to 10 fold [4]. The rice Act1 or maize Ubi1 promoters are constitutive promoters from plants that can deliver high expression of foreign genes in mononcots [5,6]. Other plant constitutive promoters have also been isolated [7-14], but few of them have been extensively used. In addition to these promoters from natural highly expressed genes, recombinant promoters designed by combining different *cis*-regulatory elements and core promoters can be useful alternatives [15,16]. For example, the pEmu promoter, which coupled the anaerobic responsive elements from the maize Adh1 gene and ocs-elements from the octopine synthase gene with a truncated maize Adh1 promoter, allowed high expression in cereal cells [17]. A G-box motif (GCCACGTGCC) tetramer fused to a CaMV -90/35S basal promoter conferred stable, strong expression in both dicot and monocot plants [18]. In another study, insertion of an amplification promoting sequence (*aps*) upstream of an expression cassette was found to increase the transcription of the adjacent heterologous genes by 2.0 to 2.5 fold in tomato and tobacco, respectively [19,20]. In addition to these efforts to promote transcription, codon optimization, proper signal localization peptide and a translation initiation sequence (GCCGCC) are also used to achieve enhanced protein translation and stability [21].

Another source of constitutive regulatory elements is cryptic gene-regulatory elements. The plant cryptic promoter tCUP conferred strong constitutive expression when fused to a reporter gene in both tobacco and *Arabidopsis*[22], and was also highly active in a wide range of dicot plants including alfalfa, canola, cauliflower, tomato and pea as well as in conifers [23-25]. The strong constitutive feasibilities of the tCUP promoter made it possible to drive the expression of a selectable marker gene for recovery of transformed plants through both organogenesis and embryogenesis [26], and proved to be a useful alternative to CaMV 35S as it does not interact with tested promoters [27]. Although the expression of tCUP is reported to be minimal in monocot plants such as wheat, barley, maize and oat [22,23,28], we considered that it would be interesting to study the possible expression and application of tCUP in rice.

Higher levels of gene expression may also be achieved through increasing the number of copies of a transgene. This may be achieved by selfing heterozygous lines [29] or crossing homozygous lines obtained from independent transformation events [30] and also by using constructs with multiple plant transcription units under the control of the same or different promoters as cited by Egelkrout et al. [1]. However, these methods are rather time-consuming and inconvenient. In this report, we demonstrate that a fragment (designated as tCUP1) derived from tCUP was active in a tissue specific manner in rice transgenic plants and behaved as a weak promoter, driving a selectable marker gene for rice transformation. Interestingly, such selection favored the recovery of transgenic plants with higher expression levels of a foreign gene controlled by a constitutive promoter, an effect ascribed to the additive result of multiple T-DNA insertions. The effect of a weak promoter driving a selectable marker on the elevation of transformation copy number and transgene expression was also confirmed by using the nopaline synthase (Nos) promoter. Thus our findings suggest a new and simple way to improve transgene expression in rice irrespective of the promoters used to control them.

## Results

### Tissue specific expression of the tCUP1 promoter in rice

**Figure 1:**
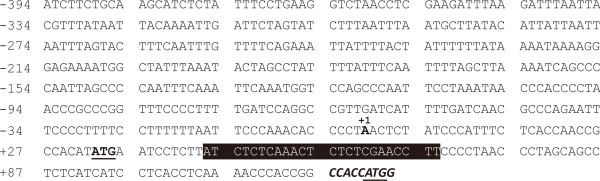
**Sequence of the tCUP1 promoter used in the experiments.** The DNA fragment between -394 and +116 of the native tCUP sequence was used as the basal promoter. The transcription start site is shown in bold and numbered with +1.The two upstream ATG codons are indicated in bold with underlining. The translational enhancer element is highlighted by a black box, a Kozak consensus sequence added at the end is shown in bold and italics. This promoter is referred to as tCUP1.

As in a previous study, the -394 tCUP truncation had similar activity to the full length tCUP promoter as shown by the 5^′^ deletion assay in *Arabidopsis*[31]. Here we synthesized a fragment named tCUP1 from -394 to +116 relative to the transcription start site of the natural tCUP promoter (Figure 1). The tCUP1 promoter was the same as EntCUP1 [23] except that the upstream translational initiation codon in the leader sequence was retained and a Kozak consensus sequence [32] including the ATG codon was introduced after +116.

**Figure 2:**
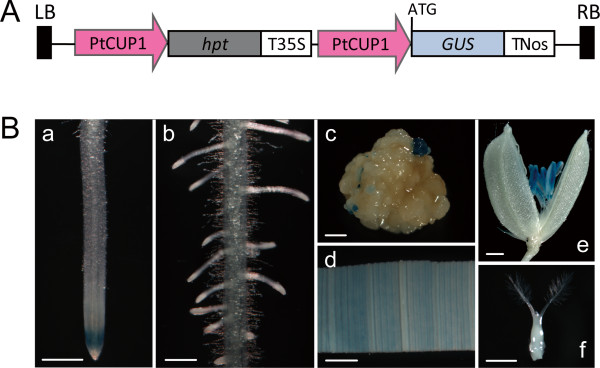
**GUS expression patterns directed by the tCUP1 promoter in transgenic rice. A**. Binary vector for tCUP1 promoter analysis which was fused with the *GUS* reporter gene and also controlled the *HPT* gene used for selection. **B.** The tCUP1 controlled *GUS* gene was expressed in transformed callus (**c**) and various organs of T_1_ transgenic rice including the root tip (**a**), leaf (**d**) and anther (**e**), but was absent in lateral roots (**b**) and the ovary (**f**). Bar=0.5 mm in (**a**) and (**b**), 1 mm in (**c**), (**d**), (**e**) and (**f**).

To determine the tCUP1 promoter activity in rice and its ability to drive selectable marker gene expression for rice transformation, the tCUP1 promoter was subcloned to a T-vector and digested for fusion respectively to the *HPT* selectable marker gene or *GUS* reporter gene in the same binary vector (Figure 2A). This vector was also designed to test whether the *HPT* expression controlled by tCUP1 can confer resistance to the usual hygromycin selection in rice. After 21-days selection on 50 mg/l hygromycin, calli were randomly selected for histochemical GUS assay. As shown in Figure 2Bc, the *GUS* gene was expressed on the primary callus showing that these cells were living after the hygromycin selection. After a second round of selection, hygromycin resistant calli were chosen for regeneration. 22 GUS positive transgenic lines were recovered and most of them showed a similar expression pattern. A representative line (Figure 2B) shows that GUS activity is mainly observed in the root tip, leaf and anthers, but is absent in lateral roots, the seed coat and ovary. This indicates that the tCUP1 promoter is expressed in a tissue specific manner in rice, unlike the constitutive expression previously observed in tobacco [23], but that it can still be successfully used for selectable marker gene expression and recovery of transgenic rice plants.

### The tCUP1 promoter is a weak but functional promoter in rice as compared with CaMV 35S

**Figure 3:**
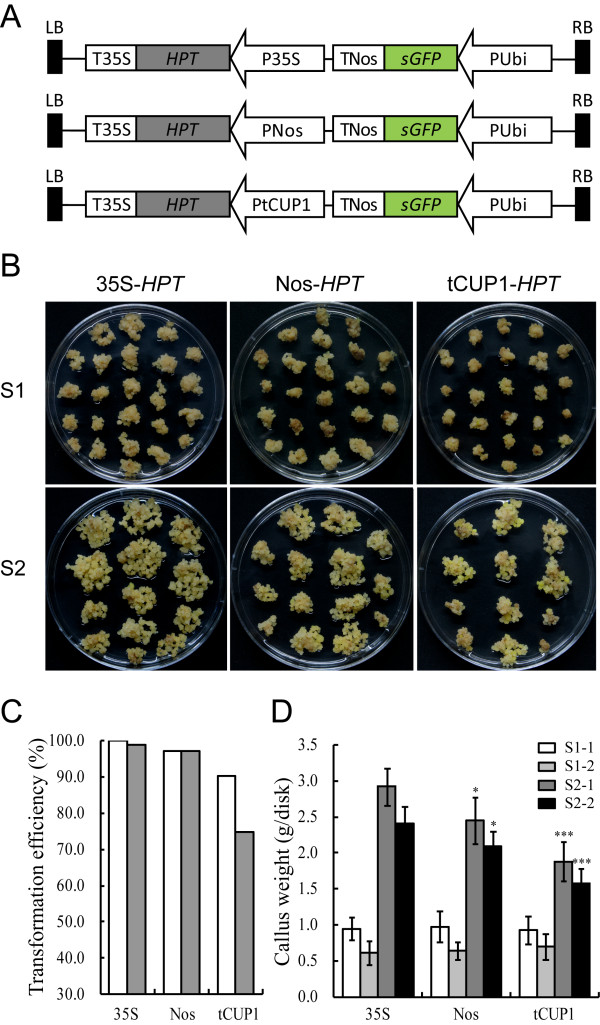
**The promoter activity of tCUP1 compared with 35S and Nos promoters. A**. Schematic diagram showing the structure of the three *HPT-GFP* vectors. These vectors were constructed based on a modified pCAMBIA0380 vector by introducing the *HPT* gene driven by 35S, Nos and tCUP1 respectively. A Ubi promoter controlled *GFP* expression cassette was also introduced into each vector as a visual indicator for successful transformation. **B.** Growth of transformed calli 21 days after the onset of first round selection on 50 mg/ml Hyg (S1) and 14 days after the onset of second round selection on fresh selection medium with the same Hyg concentration (S2). *Left panel*, transformed with 35S controlled *HPT-GFP* vector; *middle panel*, transformed with Nos controlled *HPT-GFP* vector; *right panel*, transformed with tCUP1 controlled *HPT-GFP* vector. A representative dish of calli in each transformation is shown. **C.** Transformation efficiency of the three *HPT-GFP* vectors with *HPT* regulated by 35S, Nos and tCUP1 respectively. Transformation efficiency was calculated as a percentage from the numbers of GFP expressing calli and the numbers of primary calli infected 18 days after the onset of the first round of selection. Data is shown from two independent transformations (white and gray bars) with each vector. **D.** Hyg-tolerant growth of the calli transformed by the three *HPT-GFP* vectors with *HPT* regulated by 35S, Nos and tCUP1 respectively. Weight of calli per dish was measured at the beginning of the first (S1) and second (S2) rounds of selection. Data are shown as mean ± SD (n=6). The experiments were performed twice in two independent transformations (indicated as -1 and -2) with each vector. * and *** indicate a significant difference at *P*<0.05 and 0.001 respectively, in a Student *t*-test.

We further compared the relative strength of the tCUP1 and CaMV 35S promoters in terms of their effectiveness in selectable marker gene expression and consequent tolerance to the selective agent. The double enhancer CaMV 35S promoter [3] derived from pBI121 [33] was inserted in front of the *HPT* expression cassette. Another *sGFP* expression cassette driven by the maize Ubi-1 promoter [34] was introduced within the T-DNA as a visual marker for successful transformation (Figure 3A). The tCUP1 promoter was constructed using the same strategy as CaMV 35S. The two vectors were used to transform rice calli derived from mature seeds of cultivar Nipponbare.

The percentage of calli with cells expressing GFP was determined at the end of the first round selection (S1) from two independent transformations with each vector (Figure 3C). Almost all calli (100% and 99% respectively) infected with the CaMV 35S-*HPT-GFP* vector had GFP-expressing sectors which reduced to 94.3% and 74.8% when infected with tCUP1-*HPT-GFP*. This result suggested that the transformation capacity conferred by the tCUP1 promoter mediated selection was lower than that of CaMV 35S.

Although a large proportion of the calli were transformed by the *HPT-GFP* vector controlled by tCUP1, the growth of these calli was slow, as shown in Figure 3B, whereas calli transformed using CaMV 35S grew vigorously during both the first (S1) and second (S2) rounds of selection. The weights of calli were measured at the beginning of S1 and S2. As shown in Figure 3D, there were no statistical differences at the start of S1 between the CaMV 35S and tCUP1 controlled vectors in two replicated experiments (about 0.9 mg/per dish in S1-1 and 0.6 mg/per dish in S1-2). Following S1, there were approximately 3.1 to 4.2 fold increases of weight in calli selected with the CaMV 35S vector but only 2 to 2.3 fold increases with the tCUP1 vector. The apparently low tolerance to *HPT* selection indicated that the tCUP1 promoter drives relatively weak gene expression in rice.

### The tCUP1 promoter driving low expression of the selective marker gene nevertheless provides enhanced exogenous gene expression

**Figure 4:**
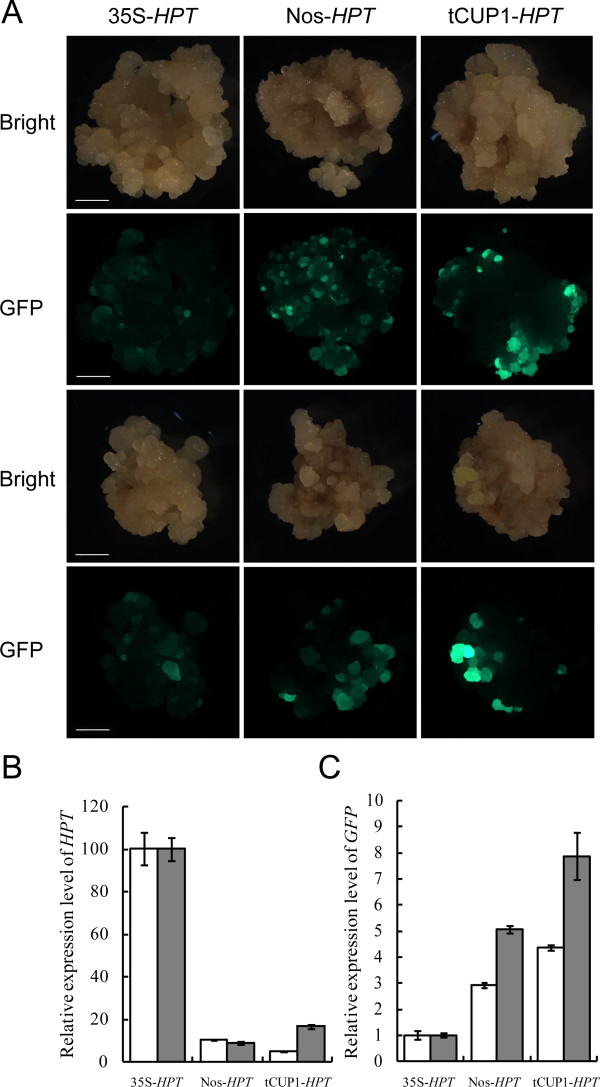
**Increased GFP expression in transformed cells when selected by a weakly expressed *HPT *gene controlled by Nos and tCUP1 promoters. A**. Images of green fluorescence emitted from callus transformed with *HPT*-*GFP* vectors controlled by 35S, Nos and tCUP1 promoters (see Figure 3A) 21 days after the onset of the first round of selection. Single representative calli from two independent transformations are shown under both bright and blue light field. Bar=1 mm. **B**. *HPT* mRNA levels in cells transformed with 35S, Nos and tCUP1 promoter controlled *HPT*-*GFP* vectors 12 or 15 days after the onset of the second round of selection. Independent secondary resistant calli were collected from 25 primary calli in each of two independent experiments (white and gray bars) for RNA extraction. Relative *HPT* gene expression levels were analyzed through qRT-PCR by normalizing to the *OsActin 1* level as a control. Data are shown as mean ± SD of three technical replicates in each independent experiment. For comparison, the *HPT* expression in the 35S vector was defined as 100%. **C.***GFP* mRNA levels in the same cells transformed with 35S, Nos and tCUP1 promoter controlled *HPT*-*GFP* vectors. For comparison, the *GFP* expression in 35S vector was defined as 1 fold.

**Figure 5:**
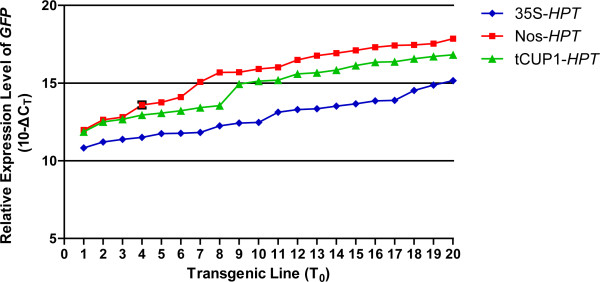
**Transcript levels of *GFP *in leaves of individual transgenic T**_**0 **_**plants with the *HPT *selectable marker gene controlled by 35S, Nos or tCUP1 promoters.** Expression levels are shown on a log scale expressed as 10-ΔCT, where ΔCT is the difference in qRT-PCR threshold cycle number between the respective gene and the reference gene *OsActin 1*. The results are the mean ± SD of three technical replicates of 20 independent T_0_ plants randomly selected in each transformation and plotted in order from low to high expression levels.

The strength of the tCUP1 promoter in regulating gene transcription was further analyzed. After the second round of selection, proliferated hygromycin resistant calli from both CaMV 35S and tCUP1 treatments were collected for RNA extraction and cDNA synthesis. The relative expression levels of *HPT* and *GFP* were analyzed by quantitative real-time RT-PCR (qRT-PCR). As expected, the *HPT* mRNA level in resistant calli controlled by tCUP1 was much lower than in those controlled by CaMV 35S (Figure 4B), correlating with their respective growth during selection. Conversely however, the relative *GFP* expression in resistant calli selected by the tCUP1 vector was found to be 4-8 times higher than in those selected by CaMV 35S from two independent experiments (Figure 4C). Similarly, the green fluorescence emitted from the resistant calli was stronger in the tCUP1 than in the CaMV 35S treatment (Figure 4A). The difference in the *GFP* transcripts between the CaMV 35S and tCUP1 vectors was also confirmed in the regenerated plantlets (T_0_) (Figure 5). A population of 20 independent lines from each treatment was randomly chosen for analyzing *GFP* transcripts in leaves. The range of *GFP* mRNA levels (shown as 10-ΔCT) in lines selected using tCUP1 was higher than in those using CaMV 35S. Differences in -ΔCT between the two highest *GFP* expressing lines containing each vector was about 1.66, and half the lines in the population selected by the tCUP1 vector had *GFP* expression levels similar to or higher than the highest one selected by CaMV 35S.

It seemed therefore that selection with a weakly expressed marker gene contributed to higher expression of the exogenous gene. To investigate this correlation, we replace the tCUP1 promoter with the nopaline synthase (Nos) promoter of *Agrobacterium tumefaciens*[35] for selectable marker expression. The Nos promoter is constitutively expressed in all tissues examined in tobacco [36], but is much weaker than the CaMV 35S full promoter in petunia [37]. There are similar differences in rice as shown in Figures 3B and 3D; the growth of calli infected with the Nos*-HPT-GFP* vector was slower than that with the vector controlled by CaMV 35S in two experimental repeats, although the transformation rates were comparable between these two promoters (Figure 3C). Furthermore, the transcriptional level of *HPT* in resistant calli selected by Nos was significant lower than in those selected by CaMV 35S (Figure 4B), while both the transcript levels and fluorescence intensity of GFP were greater (Figure 4A and 4C) as had occurred with tCUP1. Interestingly, the range of *GFP* expression levels in transgenic plants regenerated from resistant calli selected by Nos was found to be even higher than in those selected by tCUP1 (Figure 5). These data confirm that higher exogenous gene expression in transgenic rice can be reliably obtained by using weak promoters to express the selectable marker gene.

### Elevated gene expression via weak promoter selection correlates with increased T-DNA copy numbers

**Figure 6:**
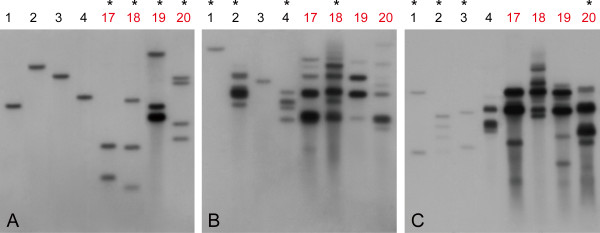
**Comparison of copy numbers among transgenic T**_**0 **_**plants transformed with *HPT-GFP *vectors controlled by the 35S, Nos or tCUP1 promoters.** Southern blot analysis of integrated T-DNA copy number in T_0_ transgenic plants transformed with 35S (**A**), Nos (**B**) and tCUP1 (**C**) promoter controlled *HPT-GFP* vectors using an *HPT* probe. DNA was extracted from leaves of 4 lines with the lowest *GFP* expression (1 to 4) and 4 lines with the highest *GFP* expression (17 to 20 in red colors) in each transformation according to the *GFP* expression analysis shown in Figure 5. About 3 μg of *Nde*I digested genomic DNA was loaded for each lane. Lines indicated with “*” were selected for further GFP observation in the T_1_ generation.

Previous work comparing different promoters driving selectable markers suggested their possible effects on the copy numbers of the transgene [38]. We therefore investigated whether the higher expression levels of the exogenous gene after using weak promoters for selection was caused by increased T-DNA copy numbers. The copy numbers of integrated T-DNA in transgenic plants produced with each of the three vectors was estimated by southern blot analysis. Lines with either the highest (line 17 to 20) or lowest (line 1 to 4) *GFP* expression were chosen from each transgenic population based on the results of qRT-PCR (Figure 5). The lines with high *GFP* expression from populations transformed using Nos or tCUP1 were found to have more hybridizing bands and stronger band signals (Figure 6B and 6C, lines 17-20) than the high *GFP* expressing lines transformed with the vector controlled by CaMV 35S (Figure 6A, lines 17-20), which varied from 2 to 4 bands. Most of the lines with low *GFP* expression from populations transformed with the vectors controlled by Nos or tCUP1 were also found to have multiple hybridizing bands (Figure 6B, lines 2 and 4; 6C, lines 1-4), but all of the low *GFP* expressing lines transformed with CaMV 35S contained only a single band (Figure 6A, lines 1-4). Based on the main hybridizing bands, there were about 2 T-DNA copies averagely integrated in lines transformed with the CaMV 35S vector, but at least 4 integrated T-DNA copies in lines transformed with the Nos and tCUP1 vectors. These results suggest that transgene expression is increased by the accumulation of multiple T-DNAs under selection with the attenuated marker gene.

The GFP fluorescence in roots of T_1_ progeny from several lines with high levels of *GFP* expression and multiple inserted copies were characterized (Additional file 1: Figure S1). Most lines were stable in GFP expression and similar in expression to their primary lines, except that line 2 (Additional file 1: Figure S1J) selected by tCUP1 controlled vector had lower GFP fluorescence than line 1 (Additional file 1: Figure S1I). The higher *GFP* expressing lines with multiple copies selected by Nos and tCUP1 promoters retained brighter GFP fluorescence in their progeny than those selected by 35S (Additional file 1: Figure S1H and S1L).

## Discussion

### The tCUP1 promoter is a functional *cis* element for rice transformation

A recent study on the tobacco cryptic constitutive promoter (tCUP) in *Arabidopsis* had shown its great ability to eliminate nonspecific interactions between transgene promoters [27]. This attractive property gave rise to the idea of applying the tCUP expression system in rice. It was reported that the tCUP sequence could direct transgene expression in both dicots and monocots, but the expression level of the reporter gene seemed lower in monocot cell suspensions than in dicot leaf tissue as measured by the numbers of GUS-positive spots [22]. Although enhanced tCUP derivatives had been used to drive the neomycin phosphotransferase II (*NPTII*) gene and selection for kanamycin resistant transformants in tobacco, cauliflower, alfalfa and *Arabidopsis*[26,27], the performance of these elements in rice was not known. We therefore examined its expression style and strength in rice and using it for selection of transformants. The two experiments were done together by fusing the tCUP1 fragment to both the *HPT* selectable marker and the *GUS* reporter. Finally, a total of 22 GUS positive lines were obtained and most of them showed similar staining patterns and inheritance to their progenies (Figure 2B). Thus it was clearly demonstrated that the tCUP1 fragment tested in this study, which comprised all three regions essential for tCUP activity in dicots [23,31], was also active as a *cis*-regulatory element in rice, although the expression was limited to certain tissues and organs. The GUS expression in transformed calli suggested that tCUP1 could mediate successful rice transformation. This was confirmed subsequently, although the selection process was prolonged because of the slow growth of resistant calli. Growth remained slow even if the upstream out-of-frame ATG was eliminated by mutation (data not shown), despite the report that this modification enhanced native tCUP activity 5 to 11 fold [23]. These results indicated that, as in other monocots, the tCUP *cis*-regulatory element was active but less effective in rice. The difference in behavior may be due to differences in transcription mechanisms between dicots and monocots, such as different transcription factors [17].

### The two effects of multiple T-DNA copy number on transgene expression

It was previously reported that an average of 2.1 copies of T-DNA were integrated in rice plants of Nipponbare when using the double CaMV 35S promoter in pCAMBIA1390 for *HPT* expression [39], which is comparable to our results using the double CaMV 35S promoter derived from pBI121. Higher transgene copy numbers are always obtained by the biolistic method, but have never been reported in transgenic rice plants produced via *Agrobacterium*-mediated methods, which is usually considered to have lower (1-5 copies) transgene copy number integration [40,41]. In our experiments, lines with extremely high numbers of T-DNA inserts (4 to 8) were frequently obtained when using Nos or tCUP1 promoters for selectable marker gene expression. In fact, these numbers could be underestimates as some of the strong hybridization signals may represent two or more inserts with a similar size migrating together as one band. Our data suggest that these multi-copy events can be screened out conditionally, for example here by attenuating *HPT* gene expression. The inadequate expression of the selectable marker under the threshold of Hyg pressure (50 mg/l) may lead to preferential survival of high copy number cells with active *HPT* expression, as apparently occurred in experiments to transform tall fescue by the PEG mediated method under elevated selection pressure from increased Hyg concentration (200 mg/l) [42]. These data indicate that the selection pressure set by both the marker gene expression level and the concentration of the corresponding selective agent is the dominant factor that influences T-DNA copy number in the resultant transgenic plants. Therefore, even if the 35S promoter is used for selectable marker gene expression, transformants with high copy number of T-DNA would be obtained when a very highly selective agent was applied.

The correlation between copy number and gene expression is complicated because multicopy T-DNA insertions are associated either with transgene silencing or with additive transgene expression depending on the nature of the inserts [43]. The negative effects of multicopy events have been extensively studied and are often discussed in relation to transformation related biotechnologies [44,45]. Generally, plants with a single copy of the transgene are preferred as they are genetically and phenotypically more stable than those with multiple copies [46,47]. In this study, we found that the transgene expression and copy number were positively correlated: high *GFP* expressers always had multiple T-DNA copies but the low *GFP* expressers had fewer; however some individuals with multiple copies had low *GFP* expression (Figure 6B and 6C, line 2 and 4). It therefore appears that multiple copies of T-DNA inserts tend to contribute to exogenous transgene expression more than to suppression or inactivation in our case, although the expression/suppression of multiple T-DNA copies varied between different studies, most likely as a result of different promoters/T-DNA insertion elements being studied, or different transformation methods used [48,49]. However, there was no linear relationship between copy number and transgene expression, perhaps because expression was also suppressed by some T-DNA inserts.

### The use of weak promoters to drive selection for effective gene expression in plants

In the study described here, we compared the effects of three different promoters in driving selectable markers for rice transformation. The constitutive CaMV 35S promoter (used as a control) yielded fast growth of callus sectors on the selection medium with 50 mg/l hygromycin, but much slower growth of calli was observed when using the tCUP1 promoter and an intermediate rate using the Nos promoter. The growth performance of the transformed calli directly suggested to us the relative strengths of these three promoters, but it was not accurate enough to determine their absolute activities, because the overall resistance depended on the number of initial transformed cells, and where and how the T-DNA(s) were inserted. We therefore tried to dissect promoter activities by comparing their transcribed *HPT* mRNA levels in transgenic lines with single copy insertion. It was an unexpected finding that the populations produced using the Nos and tCUP1 vectors were prone to have multiple insertions. This suggested to us that these promoters were too weak to produce enough resistant protein for minimal selection unless transcriptionally active insertions were accumulated. Even so, the *HPT* transcripts were still far lower than those produced by CaMV 35S (data not shown), similar to the result from resistant calli (Figure 4B). On the other hand, the increased copy number also elevated the constitutive expression of the *GFP* gene. Although it is difficult to select the homozygous line in the progeny derived from a transformant with high copy number for the purposes of genetic study, exogenous transgene expression level is still relatively stable in the next generations as we observed (Additional file 1: Figure S1). This finding provides a new strategy to select for high transgene expressers which is urgently needed for many purposes, such as the economic production of pharmaceuticals using plants as bioreactor expression systems and the generation of genetically modified plants with improved agronomic traits, especially for those vegetatively propagated plant such as potato. It will also help in the analysis of *cis*-regulatory elements when using reporter genes, particularly for those promoters with weak activities. Remarkably, the tCUP1 promoter would be a good choice for specific promoter analysis because it may also have low interaction capacity in rice.

## Conclusions

Here we report the weak activity of tCUP derived promoter (tCUP1) in transgenic rice and the application of this weak promoter in driving selection for rice transformation. The expression of a foreign gene (*GFP*) was increased both in resistant calli and regenerated plantlets, and this was caused by the large numbers of T-DNA inserts. The use of a second weak promoter (Nos) confirmed these findings. This research work suggests an effective way to maximize transgene expression and its potential applications, such as specific marker line creation and vegetative plant breeding. Our findings also suggest that care should be taken in using a weak promoter for selection if a simple insertion pattern is desired.

## Methods

### Plasmid construction

The -394 to +116 fragment of the original tCUP sequence [GenBank: AF133844] with the additional 3^′^ end Kozak consensus sequence (CCACCATGG; [32]) was synthesized commercially (Genscript, Nanjing, China). This synthesized fragment (Figure 1) was amplified with PCR primers tCUP-PF and tCUP-PR to generate the tCUP1 promoter (all PCR primer sequences are listed in Additional file 2: Table S1). The CaMV 35S and Nos promoter fragments were amplified from vector pBI121 (Clontech, Palo Alto, CA) by using PCR primers 35S-PF and 35S-PR, Nos-PF and Nos-PR respectively. All these PCR products were ligated with the pMD19-T vector (Takara, Dalian, China) for sequencing verification and then digested for cloning into the *HPT-T35S* cassette.

For acceptance of different promoters to drive *HPTII* gene expression, a promoterless *HPTII* coding region and CaMV 35S terminator fragment was amplified from pCAMBIA1300 by using primers *HPT-T35S-*F1 and *HPT-T35S-*R1. The PCR products were ligated with the pMD19-T vector (Takara, Dalian, China) and subcloned into a modified pCAMBIA0380 vector with multicloning sites (MCS) only. The resulting intermediate vector was configured with *HPT* near RB and named as *T35S-HPT-RB*. Then an *Ubi-sGFP-TNos* cassette was inserted with the Ubi promoter close to RB. Finally the 35S, Nos and tCUP1 digested fragments were cloned upstream of the *HPT-T35S* cassette to generate the three *HPT-GFP* vectors (Figure 3A).

To construct the *tCUP1*-*GUS* reporter vector, the *HPT-T35S* cassette was amplified by using primers *HPT-T35S*-F2 and *HPT-T35S-*R2 and ligated into the modified pCAMBIA0380 vector configured with *HPT* near LB and named as *LB-HPT-T35S*. A GUS (*uidA*) coding region and the nopaline synthase (Nos) terminator fragment was amplified from vector pBI101.3 (Clontech, Palo Alto, CA) by using primers GUS-F1 and TNos-R1 after the original *Sac*I site was abolished by blunt-end filling and self-ligation. Multicloning sites were added before ATG by using the GUS-MCS primer. The resulting fragment was digested and subcloned into the intermediate *LB-HPT-T35S* vector to generate the *HPT-T35S-GUS-TNos* vector. Two tCUP1 promoter fragments were ligated to *HPT* and *GUS* sequentially in fusion with its second ATG translation start site (Figure 2A).

### Plant material and transformation

The *japonica*-type rice *O.sativa* L. cv. Nipponbare was used for transformation. The binary vectors described above were introduced separately into the *Agrobacterium tumefaciens* strain EHA105 by electroporation [50]. A method of *Agrobacterium*-mediated rice transformation via scutellum-derived embryogenic calli from mature seeds was used throughout all the experiments. Dehusked mature seeds were sterilized as described previously [40] and inoculated on callus induction NB medium (Nutrient 6 macro elements, B5 micro elements and vitamins) with 2 mg/l 2,4-dichlorophenoxyacetic acid [51] at 26°C (light 12 h/dark 12 h). After 4 weeks, proliferated secondary calli derived from scutella were subcultured on fresh NB induction medium for 10-14 days before co-cultivation with *Agrobacterium*. The *Agrobacterium* strains were grown overnight in 5 ml YEP medium at 28°C and adjusted to OD_600_ between 0.1 to 0.2 by re-suspending in 30 ml liquid co-cultivation medium AAM [52] with slight modifications. For each transformation, between 150 and 200 embryogenic calli were immersed into mixed bacteria suspension cultures for 10 min, then were blot dried on sterilized filter paper for 15 min, transferred onto co-cultivation medium covered by a single sterilized filter paper and incubated for 3 days at 22°C in the dark. Calli were thoroughly washed in water with 300 mg/l carbenicillin solution, blot dried and cultured on NB medium containing 50 mg/l hygromycin and 300 mg/l cefotaxime for the first 3 weeks at 26°C (light 12 h/dark 12 h). After the first round of selection, calli were transferred to fresh NB selection medium for another 2 weeks. Weight of calli transformed with *HPT-GFP* vectors (Figure 3A) were measured per dish before and after the first round of selection to determine the callus growth activities conferred by each promoter. Hygromycin resistant calli were transferred to regeneration medium with 3 mg/l 6-benzyl aminopurine and 0.5 mg/l naphthalene acetic acid at 26°C (light 12 h/dark 12 h) for 1 month. Shoots were transferred to half-strength NB medium to obtain vigorous growth of roots.

### Histochemical GUS assay

Histochemical GUS staining was performed as described by [33]. Calli transformed with tCUP1pro:GUS (Figure 2A) 21-days after the first round of selection or tissues from T_1_ transgenic plants were collected for staining. After incubation at 37°C overnight, photos were taken through a Nikon SMZ1000 stereomicroscope equipped with a Nikon digital camera DS-Fi1. Samples stained with chlorophyll were cleared in 100% ethanol before photography. The staining solution contained 1 mM 5-bromo-4-chloro-3-indolyl-β-D-glucuronide in 0.1 M phosphate buffer (pH 7.0) with 10 mM Na_2_EDTA, 1 mM potassium ferricyanide, 1 mM potassium ferrocyanide, 20%(v/v) methyl hydrate, and 0.5%(v/v) Triton X-100.

### Fluorescence observation and photography

Images of green fluorescent protein in transformed calli 21-days after the first round of selection were visualized using a Leica M165FC fluorescent stereomicroscope and captured by a Leica DFC425C camera with the same blue light intensity and exposure time. Corresponding images in bright light were also taken. The numbers of calli expressing GFP were calculated to estimate the transformation efficiency. Images of calli growth in the whole dish after the first and second rounds of selection were taken using a SONY α550 digital camera. Images of green fluorescent protein in primary roots of 6-day-old T_1_ seedlings were observed and captured by the same fluorescent stereomicroscope and camera.

### Quantitative real-time RT-PCR analysis

After 14-days of the second round of selection, independent resistant calli were randomly collected from 25 primary calli in two independent transformations with the vectors indicated in Figure 3A. Total RNAs were extracted using Trizol regent (Invitrogen, Carlsbad, CA). 0.5 μg total RNA of each sample was subjected to cDNA synthesis using iscript cDNA Synthesis Kit (Bio-Rad, Mississauga, Ontario, Canada) according to the manufacturer’s protocol. qRT-PCR was performed with the LightCycler 480 real-time PCR system (Roche Diagnostics, Basel, Switzerland) using SsoFast EvaGreen Supermix (Bio-Rad, Mississauga, Ontario, Canada). Primers used to amplify and quantify the *HPT* and *GFP* genes are given in Additional file 2: Table S1.The relative expression levels of mRNA were normalized using the rice *Actin 1* gene (LOC_Os11g06390) and fold difference in expression was shown as 2^-ΔΔCT^. All qRT-PCR experiments were performed in technical triplicate.

For qRT-PCR analysis of *GFP* gene expression in transgenic T_0_ plants, total RNAs were isolated individually from three leaves of 20 lines randomly selected from one batch of transformation with each vector indicated in Figure 3A. Expression levels were recorded on a log scale as 10-ΔCT [53], where ΔCT is the difference in qRT-PCR threshold cycle number between the respective gene and the reference gene. Error bars are standard deviations of three technical replicates of qRT-PCR for each sample.

### Genomic DNA extraction and Southern blot analysis

Rice genomic DNA was extracted from leaves of transgenic T_0_ plants using the Sodium Dodecyl Sulphate (SDS) method according to Doyle and Doyle (1990) [54] with a few modifications. About 3 μg genomic DNA per sample was digested by *Nde*I (NEB, Beverly, MA) and hybridized with a *HPT* probe which was prepared using a random primed digoxigenin-DNA labeling kit (DIG High Prime DNA Labeling and Detection Starter Kit II, Roche Diagnostics, Basel, Switzerland) according to the manufacturer’s protocol. Primers for probe amplification are listed in Additional file 2: Table S1. Digested DNA was capillary transferred with 20×SSC onto positively charged nylon membrane (Roche Diagnostics, Basel, Switzerland) using standard protocols (Molecular Cloning III). Southern blot hybridization signals were immunologically detected with the chemiluminescence substrate CSPD and visualized by exposing to X-ray film (Kodak, Palo Alto, CA).

## Additional files

## Abbreviations

GUS: β-glucuronidase; HPT: Hygromycin phosphotransferase; GFP: Green Fluorescent Protein; Ubi: Maize ubiquitin; tCUP: Tobacco cryptic promoter; Nos: Nopaline synthase; CaMV: Cauliflower mosaic virus; aps: Amplification promoting sequence; S1: First round selection; S2: Second round of selection; qRT-PCR: Quantitative real-time RT-PCR

## Competing interests

The authors declare that they have no competing interests.

## Authors’ contributions

JZ, YY and XMW conceived the experiments. JZ, FBY and CLY carried out the Plasmid construction. JC, XMW and CY performed the RT-PCR analyses. JZ, YY and XMW carried out all the other experiments. JZ, CQY and JPC prepared the manuscript. All authors have read and approved the final manuscript.

## Supplementary Material

Additional file 1: Figure S1 GFP fluorescence in roots of T_1_ seedlings derived from transformants selected by the *HPT* gene driven by the 35S (A, B, C, D), Nos (E, F, G, H) and tCUP1 (I, J, K, L) promoters. For 35S, line 17 to 20 (A to D) were selected, for Nos, line 1 (E), 2 (F), 4 (G) and 18 (H) were selected, for tCUP1, line 1 (I), 2 (J), 3 (K) and 20 (L) were selected. Primary roots of 2 T_1_ seedlings from each of 12 lines (indicated with “*” in Figure 6), were imaged using a fluorescent stereomicroscope under blue light field with the same light intensity and exposure time. Bar= 1 mm. Click here for file

Additional file 2: Table S1 Sequences of the primers used in this study. Click here for file
