# Supplementary figures and images for: Enhanced transgene expression in rice following selection controlled by weak promoters
Source: BMC Biotechnol. 2013 Mar 27;13:29. doi: 10.1186/1472-6750-13-29 (PMC3617001; doi:10.1186/1472-6750-13-29)

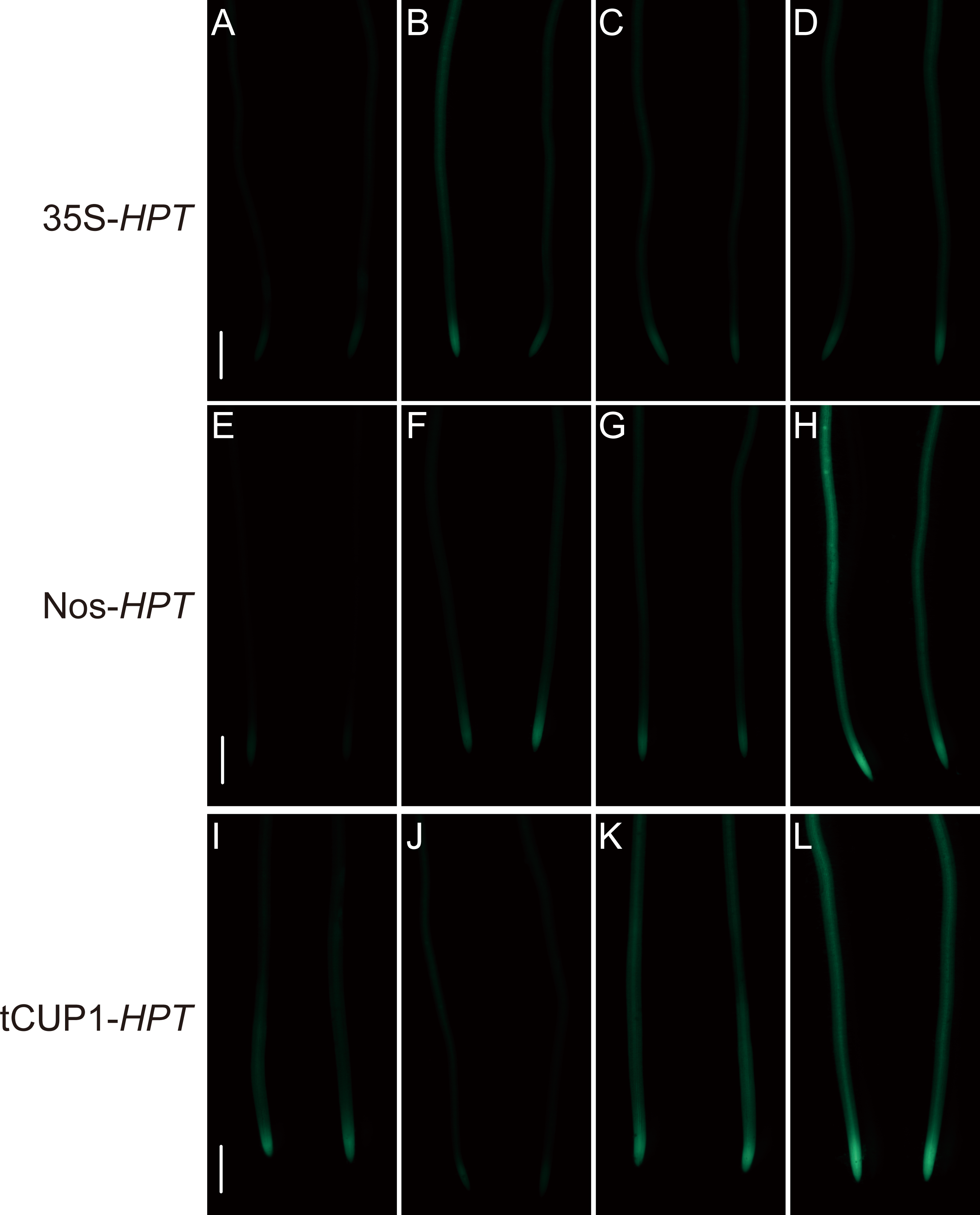

Supplement: Additional file 1: Figure S1 — GFP fluorescence in roots of T1 seedlings derived from transformants selected by the HPT gene driven by the 35S (A, B, C, D), Nos (E, F, G, H) and tCUP1 (I, J, K, L) promoters. For 35S, line 17 to 20 (A to D) were selected, for Nos, line 1 (E), 2 (F), 4 (G) and 18 (H) were selected, for tCUP1, line 1 (I), 2 (J), 3 (K) and 20 (L) were selected. Primary roots of 2 T1 seedlings from each of 12 lines (indicated with “*” in Figure 6), were imaged using a fluorescent stereomicroscope under blue light field with the same light intensity and exposure time. Bar= 1 mm. [file 1472-6750-13-29-S1.tiff]
