# Supplementary material for: Enhanced transgene expression in rice following selection controlled by weak promoters
Source: BMC Biotechnol. 2013 Mar 27;13:29. doi: 10.1186/1472-6750-13-29 (PMC3617001; doi:10.1186/1472-6750-13-29)
Supplement: Additional file 2: Table S1 — Sequences of the primers used in this study. [file 1472-6750-13-29-S2.doc]

**Table S1.** Sequences of the primers used in this study.

| Primer | Sequence (5’-3’)^a^ |
| --- | --- |
| tCUP1-PF | GGCGCGCCATCTTCTGCAAGCATCTCTATTTCC |
| tCUP1-PR | GGCGCGCCCCATGGTGGCCGGTGGGTTT |
| 35S-PF | AGGCGCGCCGGTCCCCAGATTAGCCTTTTC |
| 35S-PR | AGGCGCGCCGTCCCCCGTGTTCTCTCC |
| Nos-PF | AGGCGCGCCGATCATGAGCGGAGAAT |
| Nos-PR | AGGCGCGCCAGATCCGGTGCAGATTA |
| *HPT-T35S*-F1 | AGAGCTCGGCGCGCCATGAAAAAGCCTGAACTCAC |
| *HPT-T35S*-R1 | TACGCGTTAATTCGGGGGATCTGGATTTTAG |
| *HPT-T35S*-F2 | AGGCGCGCCATGAAAAAGCCTGAACTCAC |
| *HPT-T35S*-R2 | TGAGCTCTAATTCGGGGGATCTGGATTTTAG |
| GUS-F | ATGGTAGATCTGAGGGTAAATTTC |
| TNos-R | AAGCTTCCCGATCTAGTAACATAGATGA |
| GUS-MCS | GAGCTCCCGGGGATCCTCTAGAGTCGACGAATTCGGT  ACCATGGTAGATCTGAGGGTAA |
| *HPT*-Probe-F | ATTCCCAATACGAGGTCGCCAA |
| *HPT*-Probe-R | CTTCTACACAGCCATCGGTCCA |
| *HPT*-RT-F | CGCCGATGGTTTCTACAAAG |
| *HPT*-RT-F | ACACATGGGGATCAGCAATC |
| *GFP*-RT-F | CGACCACATGAAGCAGCACGAC |
| *GFP*-RT-R | TCCTCGATGTTGTGGCGGATCT |
| *OsAct1*-RT-F | GAGTATGATGAGTCGGGTCCAG |
| *OsAct1*-RT-R | ACACCAACAATCCCAAACAGAG |

^a^Restriction sites added at the 5’ end of primers are underlined.
